# MicroRNA/mRNA profiling and regulatory network of intracranial aneurysm

**DOI:** 10.1186/1755-8794-6-36

**Published:** 2013-09-30

**Authors:** Yugang Jiang, Mingming Zhang, Hua He, Jia Chen, Hua Zeng, Jia Li, Ranhui Duan

**Affiliations:** 1 State Key Laboratory of Medical Genetics, Central South University, Changsha, Hunan Province, China; 2 Department of Neurosurgery, Second Xiang-ya Hospital of Central South University, Changsha, Hunan Province, China

**Keywords:** Intracranial aneurysm, Microarray analysis, miRNA-mRNA analysis, Inflammation

## Abstract

**Background:**

Intracranial aneurysm (IA) is one of the most lethal forms of cerebrovascular diseases characterized by endothelial dysfunction, vascular smooth muscle cell phenotypic modulation, inflammation and consequently loss of vessel cells and extracellular matrix degradation. Besides environmental factors, genetics seem to be a very important factor in the genesis of this disease. Previous mRNA expression studies revealed a large number of differentially expressed genes between IA and control tissue. However, microRNAs (miRNA), small non-coding RNAs which are post-transcriptional regulators of gene expression, have been barely studied. Studying miRNAs could provide a hypothetical mechanism underlying rupture of IA.

**Methods:**

A microarray study was carried out to determine difference in microRNAs and mRNA between patients’ IA tissues and controls. Quantitative RT-PCR assay compared the expression level between two groups (14 IA domes vs. 14 controls) were used for validation. Validated miRNAs were analyzed using Ingenuity Pathway Analysis (IPA) to identify the networks and pathways.

**Results:**

18 miRNAs were confirmed by qPCR to be robustly down-regulated in 14 ruptured IA patients including hsa-mir-133b, hsa-mir-133a, hsa-mir-1, hsa-mir-143-3p, hsa-mir-145-3p, hsa-mir-145-5p, hsa-mir-455-5p, hsa-mir-143-5p, hsa-mir-23b-3p etc., of which 11 miRNAs are clusters: hsa-mir-1/has-mir-133a, hsa-mir-143/hsa-mir-145, hsa-mir-23b/hsa-mir-24-1, and hsa-mir-29b-2/hsa-mir-29c. 12 predicted functions were generated using IPA which showed significant associations with migration of phagocytes, proliferation of mononuclear leukocytes, cell movement of mononuclear leukocytes, cell movement of smooth muscle cells etc.

**Conclusion:**

These data support common disease mechanisms that may be under miRNA control and provide exciting directions for further investigations aimed at elucidating the miRNA mechanisms and targets that may yield new therapies for IA.

## Background

As one of the most devastating neurological conditions known to date, intracranial aneurysm (IA) has a high mortality rate and undesirable prognosis with spontaneous cerebral hemorrhage, cerebral vasospasm, and oculomotor nerve palsy as the main clinical feature. IA is common result of vascular abnormalities in the brain, with a prevalence of 3.2% in the general population, and an overall risk of rupture around 1.2% in western populations and 2.3% in Japanese series [1]. A significant proportion of aneurysmal patients are around the age of 40–60 [2,3]. Cigarette smoking, excessive alcohol consumption, hypertension and female gender are significant risk factors for IA formation and growth, and family history of IA has also been suggested to be evidence for genetic causality of cerebral aneurysms. Dysfunction of vessel cells, degeneration of vessel wall and activation of immune system were identified to be the intrinsic factors of IA development [3-6]. Its unpredictable nature and the catastrophic consequences of IA rupture remain a challenge for clinicians. Comprehensive understanding of IA pathobiology is crucial for reasonable management of IA carriers.

Due to the fact that animal models of IA are imperfect and human aneurysmal tissues are difficult to obtain, the molecular mechanisms of IA remain poorly understood. Most studies focus on mRNA expression in aneurysmal and healthy tissue to identify the alteration of gene expression within the vessel wall, which has implied some mechanisms underlying the development of IA. For example, in 2008 Krischek et al. found differentially expressed genes, which indicated that antigen processing was the most significantly associated; another study in 2009 by Shi et al. indicated that misregulated genes were mostly correlated with focal adhesion, ECM-receptor and cell communication etc. Because the large amounts of data created with each study, make a comparison or interpretation of results difficult, Roder et al. (2012) performed a meta-analysis which found seven genes showing altered expression in more than three studies: BCL2, COL1A2, COL3A1, COL5A2, CXCL12, TIMP4, TNC [7-13]. Functional studies on these genes showed that COL1A2, COL3A1, COL5A2, TIMP4, and TNC could modulate processes in the formation of the extracellular matrix (ECM), which have been described in association with IAs [10,14]. miRNA may be another layer of control in gene expression which modulates pathways and mechanisms of IA, however, expression of miRNA in IA is rarely studied.

A novel direction for IA research is the modulation of miRNA, endogenous approximately 23 nt non-coding RNAs. By binding to the 3’ UTR of complementary protein-coding mRNAs, miRNA primarily acts in the post-transcriptional repression of gene expression in animals and plants. miRNAs are incorporated into the RNA induced silencing complex (RISC) and then inhibit gene expression by either mRNA degradation or inhibiting translation which can thereby regulate up to 75% of the human genome which belong to many biological pathways including immune response and apoptosis [15-19]. Dysregulation of miRNAs have been found to have relevance to tumorigenesis, neurological, cardiovascular and developmental and other diseases [20]. Recent studies have demonstrated that miRNAs play roles in vascular remodelling and atherosclerosis [21,22]. miRNA may be another layer of control in gene expression which modulates pathways and mechanisms of IA, however, expression of miRNA in IA is rarely studied.

The role of miRNA in the molecular mechanism of IA has been of particular interest. Our study focused on investigating how the differential expression patterns of regulatory microRNAs in IA act as a potential regulator in its pathological mechanism. We generated a microRNA array followed by confirmation of miRNAs individually with qRT-PCR. We identified 18 miRNAs in 14 patients which were significantly down-regulated between IA and control tissue, 11 of these miRNAs in the cluster including hsa-mir-1/has-mir-133a, hsa-mir-143/hsa-mir-145, hsa-mir-23b/hsa-mir-24-1, hsa-mir-29b-2/hsa-mir-29c. Functional analysis indicates these miRNAs are involved with dysfunction and remolding of vascular endothelial cells, vascular smooth muscle cell and involvement of inflammatory/immune processes.

## Methods

### Patients and tissue samples

Full-thickness vessel wall samples from 14 ruptured IA domes were prospectively collected from patients (10 female, 4 male, age: 52.7 ± 8.5 ) undergoing microsurgical clipping. 14 middle meningeal artery (MMA) segments with matched sex and age were obtained during standard neurosurgical procedures (traumatic hematoma, tumor resection, IA clipping) as control. Written informed consent for participation in the study was obtained from patients. Tissue samples were snap frozen in liquid nitrogen and directly sent to the laboratory to perform RNA extraction. The collection of the human tissues was approved by Ethical Committee of the Second Xiangya Hospital of Central South, China.

### Extract RNA from samples

The total RNA was extracted by Trizol Reagent. RNA concentration and purity were determined using a NanoDrop ND-1000 spectrophotometer (NanoDrop Tech, Rockland, DE), with a 260/280 value >1.8 considered acceptable. RNA samples were further assessed for quality using a Agilent 2100 Bioanalyzer (Agilent Technologies, Foster City, CA) according to the manufacturer’s instructions to ensure an RNA integrity number > 7, and RNA samples for Agilent miRNA Chip: RIN ≥ 6.0 and 28S/18S>0.7 was used.

### Determination of specific miRNAs

miRNA microarray profile was performed using Agilent microRNA array 16.0 (3 aneurysmal wall samples and 3 healthy control samples) to identify candidate microRNAs expressed differently between patients’ IA tissues and controls. Agilent Whole Human Genome Oligo Microarray (4 × 44 K) was used for mRNA expression (2 aneurysmal wall samples and 2 healthy control samples). The microarray data can be obtained at the Gene Expression Omnibus (GEO) database (GSE46338 is the reference Series; http://www.ncbi.nlm.nih.gov/geo/).

### Confirmation of miRNA expression

miRNA and mRNA profile data were screened, keeping data with a change of more than 2 fold, then we verified the screened miRNA by RTq-PCR (SYBR® PrimeScript™ miRNA RT-PCR Kit (RR716)) according to manufacturer’s recommendation. Quantitative RT-PCR reactions were completed on CFX96™ Real-Time System. The relative expression levels of the miRNAs were calculated using the -ΔΔCT method and relative miRNA levels were normalized to U6 small non-coding RNA. We compared the expression level between two groups (14 IA domes vs. 14 controls). For the data obtained by qRT-PCR, the Mann–Whitney test and Student's t-test were used for the comparison between IA and control, and differences were considered to be significant when p < 0.05. Samples were run in triplicate and the average values were used in subsequent analysis.

### Function analysis

The selected miRNAs were further analyzed to identify the networks and pathways. For this purpose, we used software Ingenuity Pathway analysis (IPA, Ingenuity® Systems; http://www.ingenuity.com). This pathway analysis software identifies the putative targets for the input miRNA(s), integrates with our mRNA microarray profiles data, and then develops the networks and functions among the genes/targets. Before starting the analysis, miRNA targets (confidence was set to "highly predicted" and "experimental observed", species was chosen to "human") were predicted by an integrated database including miRecords, Tarbase and TargetScan Human. Then the high predicted targets were matched and paired with mRNA expression data by the expression pairing function of IPA. We assume that the expression of a given miRNA is anti-correlated with the mRNA expression of its targets. This is a widely accepted and experimentally verified supposition [23]. The results which provide us mainly with bio-functions and canonical pathways associated with our data had been generated automatically using the option of core-analysis in IPA.

## Results

### Identification of differently expressed miRNAs in IA

Focusing initially miRNA profiling data on IA tissues vs. normal tissues, there were 30 (FDR p < 0.05, ± 2 fold) differentially regulated miRNAs out of 1500 microRNAs. Among the 30 miRNAs identified, 29 were down-regulated in the IA tissue and 1 was upregulated. miRNAs identified in the microarray study were validated using individual real-time qRT-PCR assays. And 18 were found to be significantly different between the IA and control groups of the 14 patients (FC > 2 or FC < -2), and the p-value and FC were calculated (Table 1), miR-142-5p was upregulated in microarray profile, but qRT-PCR result showed no significance between IAs and controls (P = 0.25). The rest of the candidate miRNAs showed an expression tendency consistent with the array result, but without statistical significance.

**Table 1:** Network of the interactions of the miRNA target genes

**Category**	**Genes**	**Upstream miRNAs**	**Z-score/ p-value**
Migration of Phagocytes	CCL2, CCL7, CD40, CSF1, FN1, MMP14, PLAU, PLXND1, SERPINE1, SOCS3	mir-1, mir-133, mir-143-3p, mir-145-5p, mir-23a-3p, mir-28-5p, mir-455-5p	2.627/1.05E^-2^
Proliferation of Mononuclear Leukocytes	ANPEP, CD1D, CD276, CD28, CD40, CD84, CSF1, DPP4, FN1, FTL, ICOS, IL18, IL2RA, KLF4, KLRC4-KLRK1/KLRK, LILRB1, MYC, PIM1, PNP, PTPRJ, THBS1, TIGIT, TNFRSF10B, TNFRSF9, TNFSF13, ULBP2	mir-1, mir-133, mir-140-3p, mir-143-3p, mir-145-5p, mir-23a-3p, , mir-28-3p, mir-28-5p, mir29b-3p, mir-455-5p	2.612/2.66E^-6^
Cell Movement of Mononuclear Leukocytes	ADAM17, CCL2, CCL7, CD28, F11R, FN1, ICOS, IL18, ITGAL, MMP14, PDGFB, PLAU, PTPRO, RGS1, SERPINE1, SOCS3, THBS1, TLR4	mir-1, mir-133, mir-140-3p, mir-143-3p, mir-145-5p, mir-23a-3p, mir-28-3p, mir-28-5p, mir-29b-3p, mir-455-5p	2.316/2.66E^-3^
Cell Movement of Smooth Muscle cells	CCL2, CSF1, FN1, IL18, PLAU, PTGS2, THBS1, TRIB1	mir-1, mir-143-3p, mir-23a-3p, mir-28-5p	2.201/1.20E^-3^
Differentiation of Macrophages	CDC42, CSF1, LIF, MYB, PRDM1, TLR4	mir-133, mir-140-3p, mir-145-5p, mir-23a-3p, mir-28-5p, mir-29b-3p	2.166/5.70E^-3^
Stimulation of T Lymphocytes	CD28, CD40, DPP4, FN1, ICOS, IL18, KLRC4-KLRK1/KLRK1	mir-1, mir-133, mir-143-3p, mir-145-5p, mir-28-3p, mir-28-5p, mir-29-3p,	2.000/7.39E^-5^
Cell Death of Vascular Endothelial Cells	BCL2L1, IL18, LRP1, MAPK1, PMAIP1, THBS1, TNFRSF10B, TNFSF15	mir-1, mir-133, mir-143-3p, mir-143-5p, mir-23a-3p, mir-28-5p,	1.811/4.47E^-3^
Migration of Endothelial Cells	CCL2, COL4A1, DPP4, FN1, HOXA9, MMP14, NRP1, PDGFB, PIM1, PTGS2, SCARB1, SCG2, SERPINE1,TGFBR1,THBS1,TNFSF15	mir-1, mir-133, mir-143-3p, mir-145-5p, mir-23a-3p, mir-29b-3p	1.610/2.19E^-3^
Cell Movement of Endothelial Cells	ANPEP, CCL2, CDH2, COL4A1, DPP4, FN1, HOXA9, MMP14, NRP1, PDGFB, PIM1, PTGS2, SCARB1, SCG2, SERPINE1, TGFBR1, THBS1, TNFSF15	mir-1, mir-133, mir-143-3p, mir-145-5p, mir-23a-3p, mir-29b-3p	1.606/8.83E^-4^
Apoptosis of Vascular Endothelial Cells	BCL2L1, IL18, LRP1, MAPK1, PMAIP1, THBS1, TNFRSF10B	mir-1, mir-133, mir-140-3p, mir-143-3p, mir-145-5p, mir-23a-3p, mir-28-5p	1.525/1.02E^-2^
Proliferation of Smooth Muscle Cells	CCL2, FOS, MAPK1, PDGFB, SERPINE1, ST8SIA1, THBS1, TLR4, TNFAIP3, TRIB1	mir-1, mir-143-3p, mir-140-3p, mir-145-5p, mir-23a-3p, mir-28-5p, mir-29b-3p, mir-455-5p	1.037/1.83E^-3^
Proliferation of Endothelial Cells	CDH2, COL4A1, COL4A3, CSF1, DAB2, F11R, FN1, LPAR2, NRP1, PDGFB, PIM1, SCG2, TGFBR1, THBS1, TNFSF15	mir-1, mir-133, mir-140-3p, mir-143-3p, mir-145-5p, mir-23a-3p, mir-28-5p, mir-29b-3p	-1.600/3.16E^-3^

The P value indicates the likelihood of the focus genes in a network being found together as a result of random chance. Using a 99% confidence level, z-scores of 2 were considered significant. Significances for the enrichment of the genes in a network with particular biologic functions or canonical pathways were determined via right-tailed Fisher’s exact test and the whole database as a reference set. The same computation was used for gene ontology analyses of the initial gene list. The columns are arranged in descending order of z-score.

**Figure 1:**
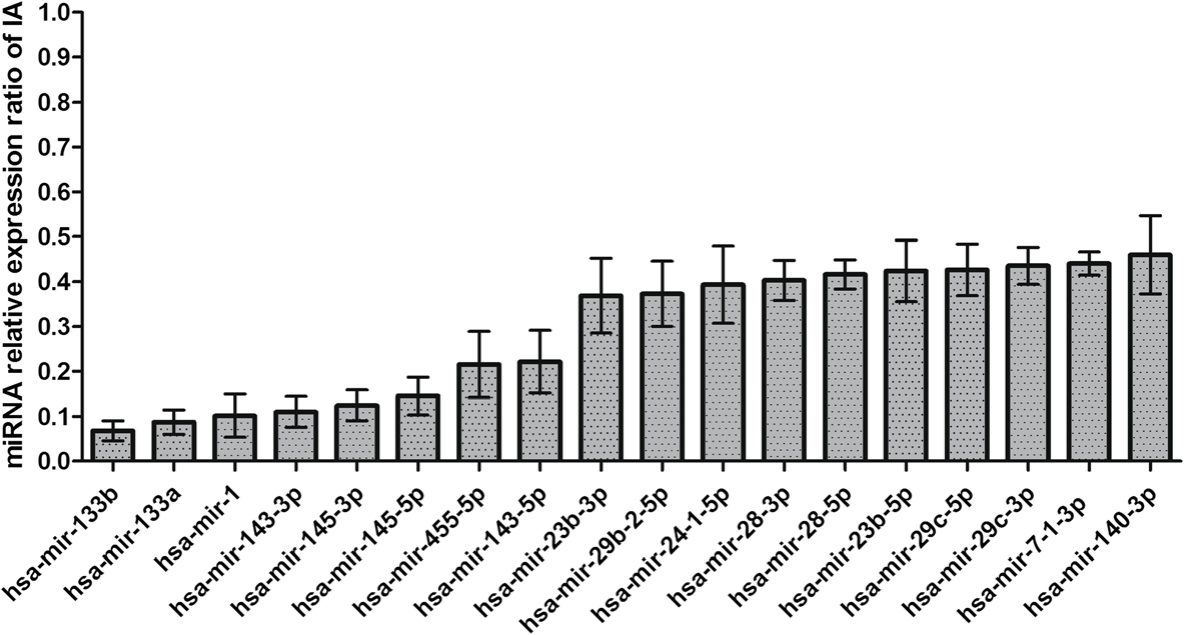
**Validation of microarray results by real-time qRT-PCR in a set of samples (n = 14).** 18 miRNAs identified as significantly different between IA and controls in the microarray study were evaluated by qRT-PCR in 14 IA and 14 control samples (MMA).

The expression levels of hsa-mir-1, hsa-mir-7-1-3p, hsa-mir-23b-5p, hsa-mir-23b-3p, hsa-mir-24-1-5p, hsa-mir-28-5p, hsa-mir-28-3p, hsa-mir-29b-2-5p, hsa-mir-29c-5p, hsa-mir-29c-3p, hsa-mir-133a, hsa-mir-133b, hsa-mir-140-3p, hsa-mir-143-5p, hsa-mir-143-3p, hsa-mir-145-5p, hsa-mir-145-3p, hsa-mir-455-5p were down-regulated at least two fold in IA compared with the control group (Figure 1). There are 4 clusters among those miRNAs: hsa-mir-1/has-mir-133a (chr18), hsa-mir-143/hsa-mir-145 (chr5), hsa-mir-23b/hsa-mir-24-1 (chr9), hsa-mir-29b-2/hsa-mir-29c (chr1).

Some miRNAs play a role in the cardiovascular system. For example, miR-1 is induced during smooth muscle cell (SMC) differentiation and increases the expression of SMC-specific contractile proteins. miR-133 is a key regulator of vascular smooth muscle cell phenotypic switch in vitro and in vivo [24]. Also notable, miR-145 is related to the thickness of the vessel wall, and the absence of miR-145 could reduce the vessel thickness and due to hypotrophy of SMCs [25]. miR-145 is down-regulated following vascular injury, during atherosclerosis, and in experimentally induced aneurysms [26,27].

### Integrated analysis of misregulated miRNAs and mRNAs

miRNAs modulate gene expression through both mRNA degradation and translational repression mechanisms, and miRNA-mRNA regulatory networks are highly complex. A dataset of 681 genes created from our mRNA microarray data paired with high predicted and experimentally observed targets to 18 miRNAs, which were used for Ingenuity Pathway Analysis (IPA). IPA results revealed top functions of these 681 common targets. The most impacted biological processes for IA including: migration of phagocytes, proliferation of mononuclear leukocytes, cell movement of mononuclear leukocytes, cell movement of smooth muscle cells, differentiation of macrophages etc. (Table 1). The functions are chosen and arranged by the z-score which indicates the predicted degree of those functions. A positive value means an increase in the function, while a negative value means a decrease the function, and the p-value indicates the significance of each function.

**Figure 2:**
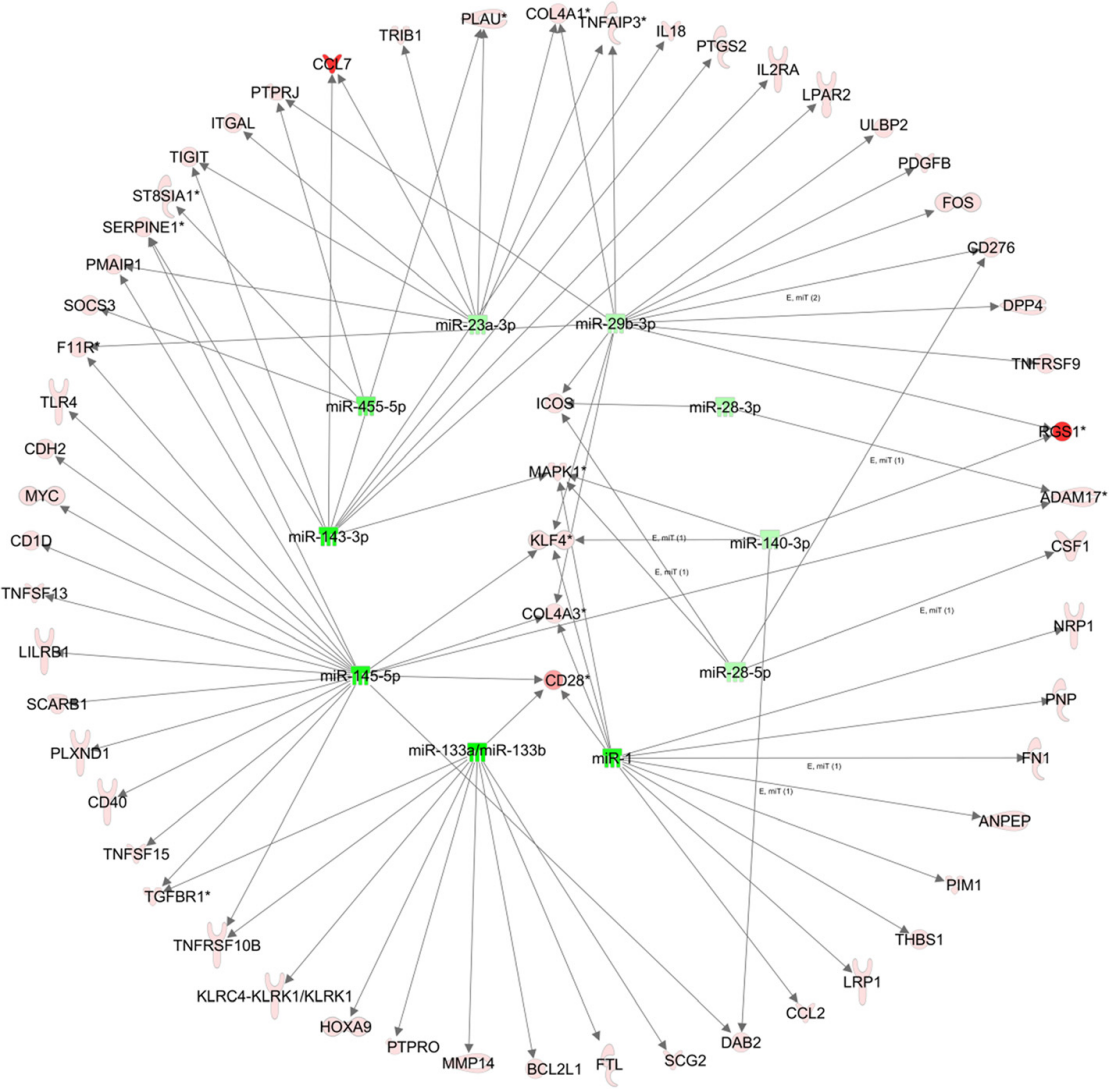
**A network of the interactions of the miRNAs and their target genes (experimental observed or highly predicted).** Ingenuity Pathway Analysis® tool was used to generate miRNA–mRNA interactions of miR-1, miR-133, miR-140-3p, miR-143-3p, miR-145-5p and, miR-23a-3p, miR-28-3p, miR-28-5p, miR-29b-3p and their targets in all selected functions. Red and green color represents the molecules to be upregulated and down-regulated respectively.

IPA predicted the most impacted biological processes for IA based on the miRNAs and their targets. 54 genes and 11 miRNAs were involved in the top 12 predicted functions, and a network generated by IPA showed the interactions between those miRNAs and mRNA (Figure 2). Some distinctive genes shown in the network targeted by more than 3 miRNAs include Kruppel-like factors 4 (KLF4); inducible T cell co-stimulator (ICOS); CD28, Mitogen-activated protein kinase 1 (MAPK1); and collagen, type IV, alpha 3 (COL4A3). Notably, among the differentially expressed mRNAs predicted to be targeted by the differential miRNAs, were some genes previously experimentally identified to be involved in aneurysm formation or loss of vessel cells, such as TGFBR1, MMPs and IL18 etc. [28,29].

KLF4 plays roles in cell proliferation, differentiation and survival. Its role, especially in the context of cancer, has been extensively studied, and several studies have explored the role of KLF4 in vascular smooth muscle cell and vascular endothelial cell. ICOS belongs to the CD28 and CTLA-4 cell-surface receptor family, and it plays an important role in cell-cell signaling, immune responses, and regulation of cell proliferation. ICOS also has been found to function in vascular diseases such as atherosclerosis [30]. CD28 is essential for T-cell proliferation and survival, cytokine production, and T-helper type-2 development. A recent study has shown that CD28 influence the atherosclerosis development by co-stimulating T-cell with CD80/86 [31]. MAPK1 is a component of MAP kinase family which is involved in a wide variety of cellular processes such as proliferation, differentiation, and transcription regulation and development. Many researchers have determined the MAPK signaling participate in the biological processes of vascular system such as proliferation of vascular endothelial cell [32] and vascular damage [33]. COL4A3 is a subunit of Type IV collagen and is the major structural component of basement membranes. Investigating these targets in this network may provide new compensatory mechanisms for protecting against IA, and may also be characterized in future studies to contribute to the miRNA-regulated response mechanisms following IA.

**Figure 3:**
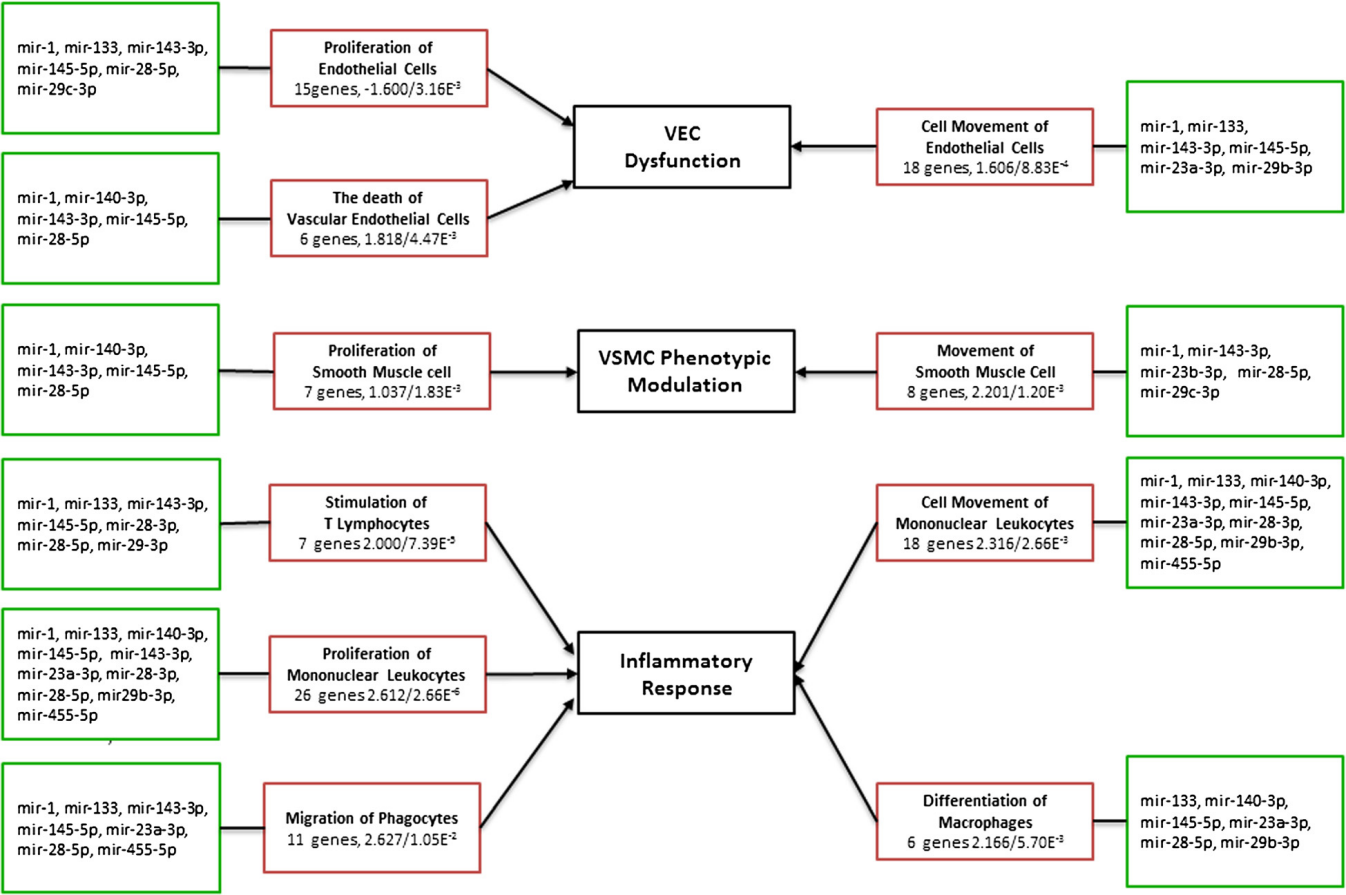
Biological categories of miRNA and predicted functions.

The mechanism that underlies the formation and development of IA is complicated and partly understood. In order to demonstrate our result more distinctively, we drew a schematic diagram to list three most impacted aspects of IA [3,5] in black box (Figure 3). Dysfunction of vascular endothelial cell (VEC), modulation of vascular smooth muscle cell (VSMC) and inflammatory response were identified to be the intrinsic factors of IA development. Several branches are connected to a red box with the name of the relevant function, genes involved in and z-score/p-value. Another green box contain the miRNAs which target the corresponding genes in the function is attached to the red one. Among those functions, migration of phagocytes has the highest z-score about 2.627, which means the function could have the maximum extent of activation based on the genes (CCL2, CCL7, CD40, CSF1, FN1, MMP14, PLAU, PLXND1, SERPINE1, SOCS3) , and those genes are validated or highly predicted targets of mir-145-5p, mir-23a-3p, mir-143-3p, mir-133, mir-28-5p, mir-1, mir-455-5p.

## Discussion

IA is the most fatal cerebrovascular system disease. Mechanisms underlying formation, progression, and rupture of IA are complex and involve a multitude of processes that are not completely understood. No safe and effective noninvasive therapies have been applied in clinical practice until recently. Treatments currently available include surgical (clipping) and endovascular (such as coiling), which have similar goals of isolating the aneurysm from blood circulation, but potentially serious complications [4]. Numerous efforts made to uncover the biology of IA have suggested that aneurysm is caused by a combination of hemodynamic stresses and defective vessel wall responses [34]. In recent years, prominent roles for microRNAs (miRNAs) have been revealed in several vascular disorders, several miRNAs have been found to be critical modulators of vascular pathologies, such as atherosclerosis, lipoprotein metabolism, inflammation, arterial remodeling, angiogenesis, smooth muscle cell regeneration, hypertension, apoptosis, neointimal hyperplasia and signal transduction pathways [35]. miRNAs may also serve as novel biomarkers and/or therapeutic targets for vascular disease [36-38]. Determining miRNA regulatory role and investigating the molecular mechanisms will expand our knowledge to better understand IA by analyzing miRNA mediated pathways.

We found that 18 miRNAs were significantly down-regulated in IA domes of 14 ruptured IA patients. There are 4 clusters among 18 miRNAs according to mirBase (http://www.mirbase.org): hsa-mir-1/has-mir-133a, hsa-mir-143/hsa-mir-145, hsa-mir-23b/hsa-mir-24-1, hsa-mir-29b-2/hsa-mira-29c. We searched the literature for information on the 18 miRNAs. miR-1, miR-133, miR-143, miR-145 are highly expressed miRs in SMCs and have been found to regulate the SMC phenotype [39-44]. miR-1 is induced during SMC differentiation and increases the expression of SMC-specific contractile proteins by targeting KLF4 [45]. Notably, the interaction which is critical for modulation of vascular smooth muscle cell phenotype, between KLF4 and miR-143/145 has also been identified [46,47]; miR-133 impairs the proliferation of SMCs and inhibits the PDGF-induced switch towards a synthetic SMC phenotype by repressing the transcription factor Sp-1 [48]. Several recent studies describe the involvement of miR-29 in aneurysm formation by post-transcriptionally repressing the expression of extracellular matrix proteins such as collagens, elastin, and fibrillins [49-54]. Several genome-wide linkage studies have determined some disease-related loci such as chr1p34.3–p36.13, chr7q11, chr19q13.3, and chrXp22 underlying the development of IA [55]. One study which is worth noting has identified several loci in familial IAs, miR-133a-1/miR-1-2 locates at chromosomes 18q11.2 which is strongly associated with the development of intracranial aneurysms [56].

miRNAs may function as provital regulators of biological processes during IA development by regulating downstream genes. A meta-analysis of five microarray gene expression studies of 60 samples revealed seven genes: BCL2, COL1A2, COL3A1, COL5A2, CXCL12, TIMP4, TNC that are very likely to be involved in the genesis of IAs [57]. These genes are also upregulated more than two fold in our samples. BCL2 is targeted by miR-143 in cervical cancer, which is involved in apoptosis and tumor formation; miR-1 regulates cardiomyocyte apoptosis by targeting BCL2 [58,59]. miR-1 is able to inhibit thyroid carcinoma cell proliferation and migration by targeting CCND2, CXCR4 and CXCL12 [60]. COL1A2, COL3A1, COL5A2 are a group of collagen genes in which mutations are associated with several connective diseases such as the involvement of COL3A1 mutations in intracranial aneurysms and Ehlers-Danlos syndrome type IV with aortic and arterial aneurysms [61,62]. miR-29 targeted several extracellular matrix genes including COL1A2, COL3A1 and COL5A2, and has been validated in nasopharyngeal carcinomas and HTM (human trabecular meshwork) cells [63,64]. Another study has observed that COL3A1 is targeted by miR-29 as a participant in the mechanism of atrial fibrillation [65].

A break in the delicate balance between local hemodynamic stress and arterial wall integrity may be the reason why IA occurs. Genesis of IA may be triggered by aberrant flow conditions, and a group of activated cells could lead to an unstable situation between "repair and maintain" and "degrade and destroy", following which dysfunction of endothelial cell, and loss of mural cell and inflammatory response may eventually lead IA rupture [3]. Lots of cells and genes are abnormally modulated during the development of IA, investigating those miRNA or mRNA found in our study and their regulating networks may provide new insight of IA pathogenesis.

Analysis generated by IPA on those validated miRNAs and their putative targets revealed that these miRNAs may be involved in the three main pathological processes: loss of vessel cells, phenotypic change of vessel cells, and inflammation of the vessel. Several targets of miRNAs have been reported to function in the loss of vessel cells which is the main characteristic of the late stages of the human aneurysmal disease. For example, Thrombospondin 1 (THBS1), also known as TSP-1, targeted by miR-1 [66], and form a subunit of a disulfide-linked homotrimeric protein. Study of human aortic smooth muscle cells (HASMC) has revealed that TSP-1 is involved in the migration and proliferation of HASMC, moreover, the upregulation of TSP-1 by leptin is depended on JAK2 and MAPK pathways [67]. Activated movement of smooth muscle cell and migration/movement of endothelial cell may imply phenotypic modulation of those cells. Neuropilin 1 (NRP1), validated to be targeted by miR-1 [66], participates in several different types of signaling pathways that control cell migration, for example, NRP1 binding with VEGF is essential for stimulation of endothelial cell migration [68]. One of the crucial players in the pathophysiology of IA is inflammation. Some studies at the transcriptome level are in accordance with the histopathological series that associated endothelial dysfunction, loss of mural cells, inflammatory cell infiltration and degradation of the matrix with sIA wall rupture [69].Though inflammatory macrophages and lymphocytes infiltrate the aneurysm wall, a link between their presence and aneurysm growth with subsequent rupture is not completely understood [70]. 5 out of 12 functions are related to immune response, among those functions, migration of phagocytes, affected by 7 miRNAs and 11 genes, has the highest potential to be activated. Genes validated to interact with miRNA include PNP, MYC, CD276, PIM1, THBS1, F11R and PLAU etc. which are involved in immune response. For example, purine nucleoside phosphorylase (PNP) targeted by miR-1 [71], is associated with T-cell (cell-mediated) immunity, B-cell immunity and antibody responses [72].

Functional analysis revealed some molecules targeted miRNAs with high prediction, validation of the relationship between the miRNA and these predicted targets are necessary for extending the molecular network of IA. Some upregulated genes expressed in intracranial arteries including NLR family, tumor necrosis factor (ligand) superfamily, interleukin, fibronectin and chemokine are predicted targets of down-regulated miRNAs in our study. Previous studies have implied their importance in IA [11,57]. NLRP1, predicted target of miR-143-3p, is a member of the Ced-4 family of apoptosis proteins that could induce caspase-1 activation through the assembly of inflammasomes, multiprotein complexes, which are critical for generating mature proinflammatory cytokines including IL-1β and IL18. IL18 is also a predicted target of miR-143-3p, which increase early stage apoptosis of cultured HUVEC (umbilical vein endothelial cells) cells [73], and increase the death of VEC [74]. Another apoptosis related gene is TNFSF15, predicted target of miR-145-5p, which belongs to the tumor necrosis factor (TNF) ligand family acts as an autocrine factor to induce apoptosis in endothelial cells by activating NF-kappaB and MAP kinases [75]. FN1, targeted by miR-1, is involved in cell adhesion and migration processes. Two cytokine, CCL2 and CCL7, were targeted by miR-1, miR-23a-3p and miR-143-3p respectively. CCL2 displays chemotactic activity for monocytes and basophils which has been implicated in the pathogenesis of atherosclerosis which is characterized by monocytic infiltrates [76]. CCL7 is a secreted chemokine which attracts macrophages during inflammation and metastasis [77]. Although their interactions with miRNAs are predicted, validation the interactions in IA tissue could unearth the pivotal role of miRNAs in the pathogenesis of IA.

### Limitation

This study has several limitations. One limitation is that only end-stage of disease tissues are available, as only the human IA samples are large enough or ruptured and need surgical intervention can be obtained. IA dome contains different cell type and tissues, the contribution of misregulated genes in each cell type should be further determined. Our functional analyses were based on miRNA targets which include highly predicted and experimentally validated, so these highly predicted interactions should be validated in future research.

## Conclusion

Our data clearly showed the differential expression of 18 miRNAs in IA tissue from a control group of human MMA tissue. Bio-informatic analysis by IPA indicates that miRNAs target genes which may play a role in functional changes in VEC and VSMC, and activation of inflammatory response, and loss of cells in vessel wall. Our study was in line with many previous studies, several candidates need to be studied thoroughly to uncover the role of miRNA in IA. Our study provides novel evidence identifying miRNAs involved with response of the rupture of IA and gives us a deeper understanding on pathology of IA, miRNA found in this study may be a notably potential entry point to reveal pathology of IA from another perspective.

## Competing interests

The authors declare that they have no competing interests.

## Authors’ contributions

YJ and MZ recruited patients, obtained tissue samples from cases and controls, verified clinical information, designed experiment, analyzed data, and critically reviewed the manuscript. HH, JC and HZ prepared RNA samples for microarray and RTq-PCR, and ran the RTq-PCR assay. JL contributed to the microarray data handling, functional analysis, as well as drafting and editing the manuscript. RD contributed to the experimental design, data analysis, drafting and editing of the manuscript, and obtained funding for the study. All authors read and approved the final manuscript.

## Pre-publication history

The pre-publication history for this paper can be accessed here:

http://www.biomedcentral.com/1755-8794/6/36/prepub
